# A multi-task deep learning approach for landslide displacement prediction with applications in early warning systems

**DOI:** 10.1038/s41598-025-29084-1

**Published:** 2025-12-08

**Authors:** Damjan Strnad, Domen Mongus, Štefan Horvat, Ela Šegina

**Affiliations:** 1 Faculty of Electrical Engineering and Computer Science, University of Maribor, Koroška cesta 46, SI-2000 Maribor, Slovenia; 2 Geological Survey of Slovenia, Dimičeva ulica 14, SI-1000 Ljubljana, Slovenia

**Keywords:** Landslide displacement prediction, Neural network, Multi-task learning, Landslide early warning system, Remote sensing, GNSS, Natural hazards, Computer science

## Abstract

Accurate landslide displacement prediction is important for the construction of reliable landslide early warning systems (LEWS). Recently, deep neural networks have become the dominant approach for landslide displacement modeling. However, we show that focusing solely on low prediction residuals is not perfectly aligned with the goals of LEWS, where the emphasis is on precise forecasts near the warning threshold. This can result in poor efficiency of threshold-based warning prediction. We propose a multi-task approach to model training, where auxiliary targets are used to optimize the model towards the performance relevant for LEWS. The methodology is validated using the data from the deep-seated Urbas landslide in north-western Slovenia, which has been monitored by GNSS since 2019. Developing a displacement prediction model for Urbas is a step towards extending the existing wire-based mechanical alarm system. We employ a convolutional neural network for day-ahead displacement prediction using recent landslide activity, hydrometeorological measurements and seismological data. The proposed multi-task model retains a competitive $F_1$ score for warning prediction while achieving a significantly lower mean absolute error compared to the reference models. The proposed methodology is generally applicable and has the potential to improve the efficiency of landslide modeling in the context of LEWS.

## Introduction

Landslides are highly destructive natural disasters causing material damage and claiming numerous human lives every year world-wide^1^. By analyzing the emergence and progression of existing landslides, preventive strategies can be devised to minimize risks to human life and remediation costs^2,3^. In recent years, a wider adoption of machine learning (ML) techniques in landslide research can be observed. One of the primary applications of ML in landslide studies is temporal landslide prediction^4^, which deals with determining the likely time of slope failure or intensification of landslide activity^5^. It relies on the monitoring of an existing landslide and building a model of its dynamics based on external factors, such as rainfall and groundwater level (GWL). Such model can be used to predict future landslide behavior given the anticipated values of these factors.

Landslide hazard prediction is a fundamental component of modern landslide early warning systems (LEWS), which allow timely evacuation of population from areas where adequate protective actions are not possible or economically reasonable^6^. The main indicators of increased landslide risk employed by such systems are the expected duration, amount and intensity of precipitation^7^. The rainfall threshold values for triggering landslide alerts are determined statistically from historical observations, or derived using physical simulation models^8^. In combination with landslide susceptibility maps, such systems can be used as general warning systems on a local^9^ or regional^10^ scale.

LEWS can also be designed to indicate the expected increase of activity in slow-moving, deep-seated landslides, which pose ongoing and persistent threats^11^. They can be monitored continuously using on-site sensors and remote sensing techniques such as Global Navigation Satellite Systems (GNSS), which provide high spatio-temporal resolution of data^12,13^. This allows one to build a more accurate model of landslide dynamics, and use it to predict the expected landslide behavior under various future conditions. Landslide displacement analysis is pivotal to reliable prediction of landslide acceleration conditions which could trigger catastrophic events. Besides enabling higher precision of forecasting, tailored to a particular landslide, such models can be used to gain valuable insights about the interplay of geological, hydrological and meteorological factors that affect landslide dynamics^14^.

The fusion of remote sensing and ML techniques has contributed to recent advancements in landslide modeling^15^. Deep learning models, such as neural networks, are capable of capturing non-linear relationships and complex patterns that underly landslide dynamics^16^. However, careful preparation of training data, selection of model architecture and tuning of training parameters are required. Most of the existing studies in landslide displacement prediction use the data from the Three Gorges Reservoir Area (TGRA) in China^17^, where several landslides were equipped with remote sensing systems for monitoring.

The progress in ML-based landslide displacement prediction has largely followed the broader development of ML model architectures. An early attempt by Du et al. ^18^ used a feedforward neural network to predict the periodic displacement component of two TGRA landslides, while Lian et al. ^19^ employed extreme learning machines (ELM) to predict both the trend and the periodic displacement. A variant of ELM performed best in a displacement prediction comparison by Deng et al. ^20^, who used acoustic emission measurements and rainfall data as inputs. Hybrid solutions involving signal decomposition techniques and ML models have also been successfully applied in displacement prediction^21^.

Significant advancement in ML-based displacement prediction was achieved with the introduction of recurrent neural networks^22^, such as LSTM^23–25^ and GRU^24,26^, which can capture the temporal patterns of landslide dynamics. Zhang et al. ^26^ used the monthly rainfall, average reservoir water level and its changes as inputs to GRU for monthly displacement prediction of the Jiuxianping landslide, while Liu et al. ^24^ compared the performance of LSTM and GRU with mixed results.

The next evolutionary step in landslide displacement modeling was a combination of recurrent and convolutional neural networks (CNN). A hybrid CNN-LSTM was used by Li et al. ^27^ to predict the periodic and random displacement components, demonstrating the superior performance of hybrid architecture over plain LSTM and GRU. Similar conclusions were reached by Lin et al. in a study addressing monthly displacement prediction^28^, where a CNN was used for local feature extraction and a bidirectional LSTM for modeling the long-term temporal dependencies. Wang et al. ^29^ enhanced the CNN-LSTM model with a biological growth model to further improve the prediction accuracy.

The latest generation of models incorporates the attention mechanism^30^. An attention-based CNN-LSTM model was proposed by Yang et al. ^31^ for daily spatio-temporal displacement prediction using reservoir water levels, rainfall, and landslide deformation measurements from six monitoring stations. The proposed model produced better predictions than the plain LSTM and the CNN-LSTM model without the attention mechanism in several cases, but it did not consistently outperform them. Xi et al. ^32^ conducted a comparative study by evaluating different machine learning models for daily displacement prediction at seven measurement points. It was shown that the attention-based models outperformed other models in five cases. Meng et al. ^33^ similarly found that extending the hybrid CNN-BiGRU model with an attention layer improved the detection of local displacement peaks. Recently, Ge et al. ^34^ showed that efficient displacement prediction can be achieved by replacing the convolutional and recurrent layers with Transformer encoders and decoders.

Landslide displacement prediction research involving locations outside TGRA is rather sparse. In order to predict daily displacements of the Laowuji landslide, Xie et al. ^23^ considered both natural factors and human activities and used LSTM to model the periodic component of the displacement. In another study, Nava et al. ^35^ compared several neural network models for daily displacement forecasting of landslides inside and outside the artificial reservoir context. The inputs were previous landslide movements and rainfall; for landslides within the artificial reservoir context, the water level height was also given. The results showed that a multi-layer perceptron (MLP), LSTM and GRU produced reliable predictions in all scenarios, while the combined convolutional and LSTM architecture was the best for highly seasonal landslides. The latter is consistent with the conclusions of TGRA studies, since TGRA landslides typically exhibit significant periodic component. Kuang et al. used a combination of graph convolutional neural network and spatially local transformer^36^ to capture dynamic spatio-temporal correlations among thousands of monitoring locations.

Despite significant progress in ML-based landslide displacement prediction, several challenges remain. These include the limited quality and availability of monitoring data^37^, difficulties in identifying of relevant predictors and temporal correlations^38^, and expansion of models from slope scales to regional scales^39^. The integration of physics-based and data-driven approaches into hybrid models^39^ and achieving the robustness required for model incorporation into LEWS^35^ also remain open challenges.

Most existing research on ML-based landslide displacement prediction focuses on reservoir-based landslides^40^, where the task is to accurately model the periodic displacement component driven by seasonal rainfall and reservoir water level fluctuations^41^. The forecasts are typically issued on a monthly scale, which limits their usefulness for early warning applications. On the other hand, there is a lack of experimental studies on ML-based daily prediction of non-reservoir landslides, particularly regarding their potential usefulness for LEWS.

In this paper, we present a methodology used for daily landslide displacement prediction of a slow-moving alpine Urbas landslide in north-western Slovenia^42^. The landslide lies on the mountain slopes and threatens to develop into a massive debris flow with devastating consequences for the settlement Koroška Bela below. Modeling the dynamics of Urbas landslide is therefore important for the implementation of an early warning system based on daily predictions. To this end, a near real-time GNSS-based monitoring system for surface movement has been installed, and a network of measurement equipment deployed in the landslide area in 2019^43^. It was shown in a previous study that the dynamics of Urbas landslide are strongly influenced by hydrometeorological conditions, in particular antecedent average daily precipitation^44^. However, the analysis was limited by short monitoring duration (less than two years) and a lack of significant meteorological events during the observed period.

The present study presents the first systematic attempt to train an ML-based data-driven displacement prediction model for the Urbas landslide. In this study, a neural network model is trained to predict daily landslide displacements from recent movements, hydrometeorological conditions, and seismic events. The proposed methodology employs a multi-task learning approach to improve the model performance for its prospective use in a threshold-based LEWS. The training data are obtained as hourly, daily, or non-uniform time series from a variety of heterogeneous sources, including sensors installed on the landslide, satellite observations, and publicly available data archives maintained by the Slovenian environment agency^45^.

The main contributions of this study are:

- it proposes a multi-task approach to neural network training for landslide displacement prediction, which uses auxiliary learning objectives to improve the prediction of landslide mobilization events,
- it analyzes the aspects relevant for incorporating the model in a short-term LEWS, such as threshold activity detection and the impact of inacurate weather forecasts on model performance,
- it explicitly addresses the problem of missing data and outliers, which is unavoidable part of the in-situ monitoring, but is rarely discussed in related works.

## Methodology

### Study area

**Fig. 1:**
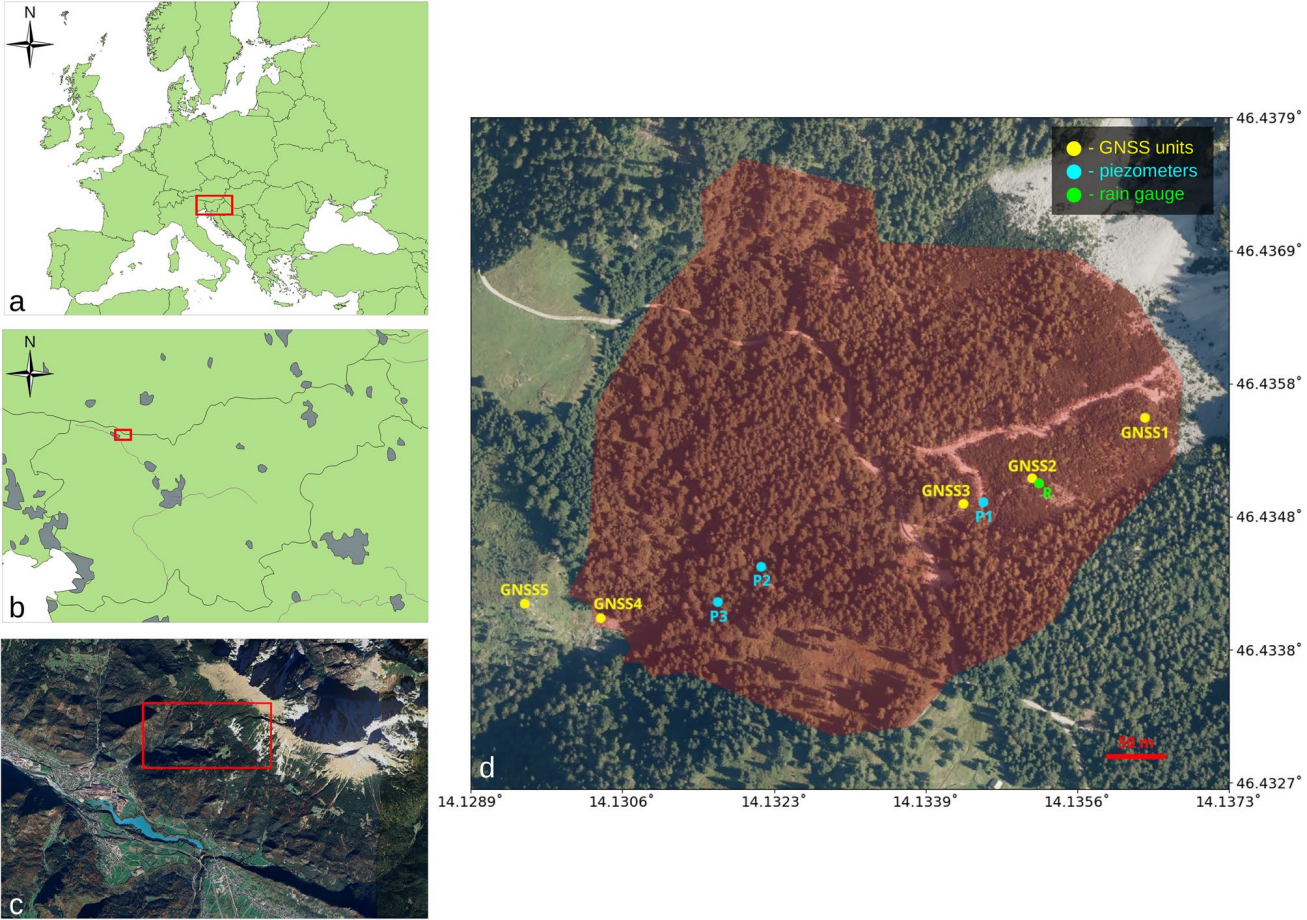
The location of the study area in **a** Europe, **b** Slovenia, and **c** Koroška Bela region, and **d** The landslide area with marked locations of on-site GNSS units (yellow dots), piezometers (blue dots) and a stationary rain gauge (green dot). The figure was created using QGIS (version 3.34) and LibreOffice Draw (version v24.2).

The subject of this study is the Urbas landslide, which resides on the southwest-facing slopes of the Karavanke mountain chain in north-western Slovenia (Fig. 1). It is one of the largest landslides in Slovenia and an active part of unstable hinterland of Koroška Bela, a settlement with approximately 2,200 inhabitants, which has a long history of debris-flow events^46^. The landslide covers an area of 177,000 m$^2$, with the maximum volume of the sliding mass estimated at 1.5 million m$^3$^47^. The median elevation of landslide body is approximately 1250 m above sea level, and the slope angles on the site range from 30$^\circ$ to 70$^\circ$^43^.

Urbas landslide formed in a several hundred meters wide area of soft, fine-grained tectonically deformed Upper Carboniferous to Permian clastic rocks consisting of claystone, siltstone, sandstone and conglomerate, that stretches along the central part of slopes in the wider area of Karavanke mountain range^46^. The landslide formed within the Košuta fault zone dissected by several NW-SE faults^48^. The stretch of Palaeozoic clastic rocks is up the slope limited by the Mesozoic carbonates and downslope by the Palaeozoic and Triassic carbonate rocks^46^. Clastic rocks are partly covered with scree material originating from the rockfall area that formed at the contact with the Mesozoic carbonates. The lower part of the landslide is characterized by mixed gravel, soil and debris that is exposed to intensive surface erosion driven by the stream that drains the landslide area. Continuous outwash of the material disables the deposition of a typical landslide foot. Instead, paleo alluvial deposits indicate that the landslide material mobilized into the debris flow that reached the bottom of the valley^48^. For detailed geological and tectonic descriptions of the Urbas landslide, the reader is referred to Peternel et al.^46^.

**Fig. 2:**
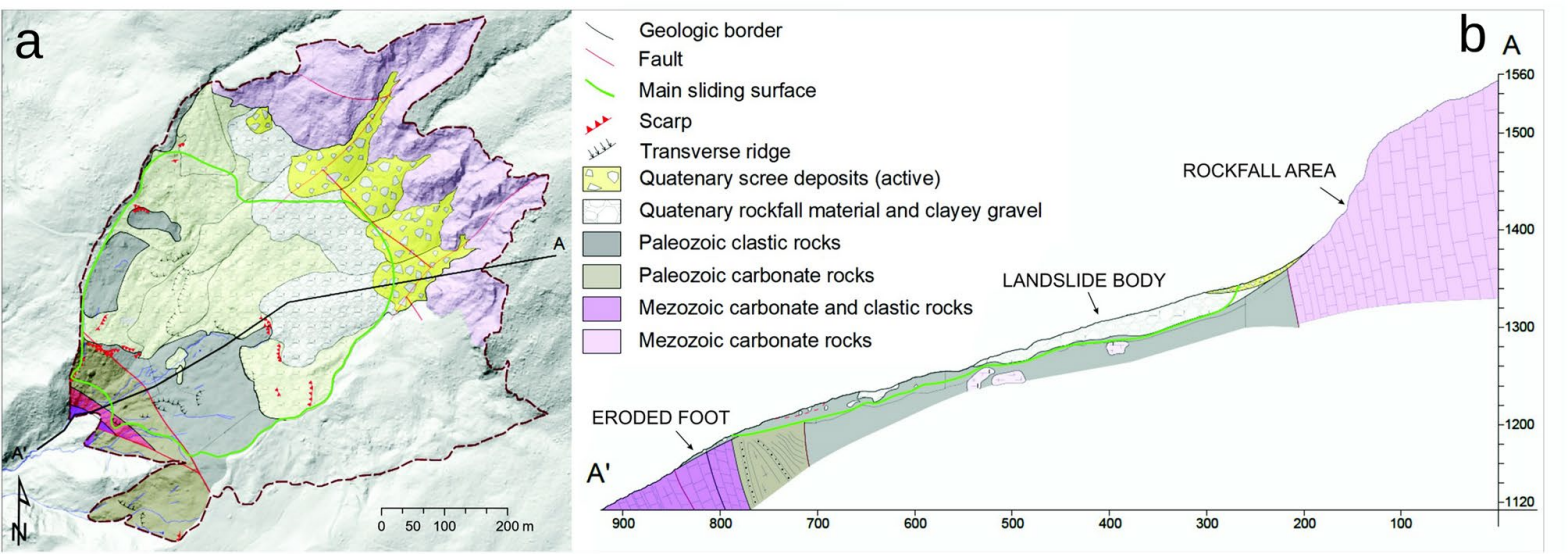
**a** Geologic map and **b** geologic profile of the Urbas landslide (adapted from Šegina et al.^42^, CC BY).

Following Cruden & Varnes^49^ classification, the Urbas landslide is a composite landslide characterized by three different mechanisms of displacement: rockfall in the upper part, deep-seated sliding in the central part and debris-flow in the lower part of the composite landslide^42^. In terms of the sliding kinematics, recent study indicates translational type of landslide movement. Such sliding pattern is characteristic of the upper and central part of the landslide body. The central part of the landslide body moves continuously at low sliding rates (0.2 mm/day on average, 330 mm in 5 years). The displacement is induced by up to 20 m thick scree accumulation under the rockfall area acting as an additional load on the uppermost part of the landslide body^42^. In the lower part of the landslide, the steady movement is locally accelerated due to the topographical narrowing of the space available for the transport of the material through the debris-flow. In addition, occasionally increased displacement rates are related to changes in external factors. This is the most active part of the landslide with cumulative displacements of 2780 mm in 5 years and recorded rates of up to several cm/day. Due to its sensitivity to external factors, high activity and high risk for mobilization to debris flow, the monitoring point at this location was selected for modelling landslide displacement prediction presented in this paper (Fig. 2).

The first monitoring of Urbas landslide was performed in 2011 using InSAR and GNSS measurements. It discovered a relatively large horizontal displacement of 32 mm over a short period of six months. Continuous monitoring of the landslide began in 2019, when a low-cost GNSS system was deployed to the site^44^. Four self-sufficient GNSS units (GNSS1-4), powered by solar panels, were set up at fixed locations on the landslide, with an additional local reference unit GNSS5 mounted outside the landslide area (Fig. 1). Three of the GNSS units (GNSS1-3) measure cumulative displacements in the top part of the slide, which shows consistent trend with little indication of influence from external triggers. The remaining unit GNSS4 is located at the toe of the landslide, where the landslide dynamics are significantly more active^44^. Because the evolution of the landslide toe is also more important from the perspective of early hazard detection, the displacement time series at GNSS4 is used as the modeling target in this paper.

The GNSS system supplemented the pre-existing in-situ hydrogeological observation system consisting of inclinometers, piezometers, and rain gauges. The inclinometers provide insights into the subsurface layering of movements in the main body of the landslide. They are not used for the modeling of surface displacements, but enable determining the sliding depth and assessing the geomechanical properties of the landslide^44^. The piezometers are used to monitor GWL along the central longitudinal landslide axis. The groundwater table is recharged by infiltration of rainfall and melting snow through the upper soil layers. It resurfaces in the lower part of the landslide, where the water flow from the springs causes strong surface erosion and mobilizes downstream deposition of weathered material^44^. To record local precipitation data, a rain gauge was installed on the Urbas landslide. A broader range of valuable weather data, including snow depth and temperature, is collected by nearby weather and precipitation stations. One of them is positioned on a smaller Čikla landslide, approximately 500 m west of Urbas.

In August 2023, the mobilization of material from the landslide that cut the existing wire-based mechanical alarm system led to evacuation of the inhabitants in the valley. It was clear that besides alarming in case of emergency, a reliable prediction model that would help at decision-making in case of interventions, is necessary.

### Data sources and preprocessing

This study deals with displacement prediction of the lower part of Urbas landslide, which is the most active part of the landslide and a potential source of debris flow that presents direct hazard to the population of Koroška Bela. The displacement data are being collected on the landslide since 2019, where the GNSS measurements at an observation rate of five seconds are processed into daily displacement values using specialized software. The standard deviation of GNSS4 measurements, computed after removing the main trend using a 7-day spline model, is $\sim$1.5 mm in the planimetric direction and $\sim$2 mm in the vertical direction. The technical details of GNSS monitoring system on the Urbas landslide can be found in the paper by Šegina et al. ^43^.

For the purposes of modeling the Urbas dynamics, the following monitored hydrometeorological parameters were considered as potentially influental covariates in this study:

- the amount of rainfall,
- groundwater level,
- snow depth, and
- air temperature.

Previous studies have found that daily displacements showed moderate to strong correlation with prolonged periods of antecedent precipitation and fluctuations in the groundwater table^43,44^. These two indicators are partially correlated because the groundwater table is replenished by rainfall infiltrating through the upper soil layers, but there is a more complex relationship between the GWL and hydrological conditions in the drainage basin of the landslide area. The study by Peternel et al. further indicated that the influence of snowmelt should also be considered^44^. While the rate of snowmelt depends on many environmental factors, we use snow depth and air temperature as the only currently available indicators of the amount of released water.

The hinterland of Koroška Bela is characterized by complex geological and tectonic conditions, which lead to high slope instability^46^. Seismic activity within a wider region can consequently influence the landslide dynamics. The data about earthquakes, which are detected by the country’s seismographic network, are reported by the Slovenian Environment Agency on a monthly basis. The earthquake magnitude, depth, and distance of epicenter from the landslide were considered as additional inputs to the prediction model in this study.

Table 1 summarizes the main properties of all data sources used in the study. The data from different sources were available at different observation rates; some of the time series contained erroneous readings and gaps. These issues were resolved with data preprocessing, which involved cleaning, imputation, and temporal alignment of the time series. Some parameters were measured by multiple on-site sensors, which allowed imputaton of missing data by modeling the cross-correlations between related time series. In other cases, publicly available data from nearby weather stations were used for imputation. Temporal alignment of covariates to full hours was achieved by interpolation of data points when necessary. The hourly data were finally aggregated into daily time series, where the aggregation method depended on the type of the data. The outputs of the preprocessing step were temporally aligned time series of covariates at daily resolution. In the following subsections, we provide details about the data preparation process for individual time series.

**Table 1:** List of data sources and their properties.

Data type	Source	Resolution	Missing data
Landslide displacements	On-site GNSS units	Daily	Yes
Rainfall	On-site rain gauges	Hourly	Yes
Groundwater	On-site piezometers	Hourly	Yes
Snow depth	Nearby weather stations	Daily	Yes
Temperature	Nearby weather stations	Hourly	Yes
Earthquakes	National env. agency archive	Time points	No

#### Displacements

**Fig. 3:**
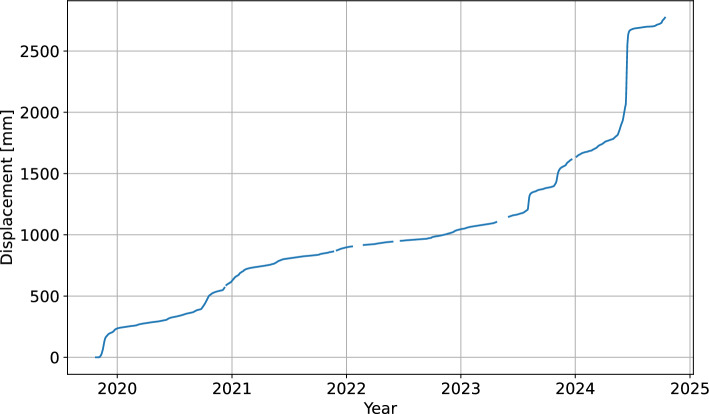
Time series of cumulative displacement for the bottom part of the Urbas landslide.

**Fig. 4:**
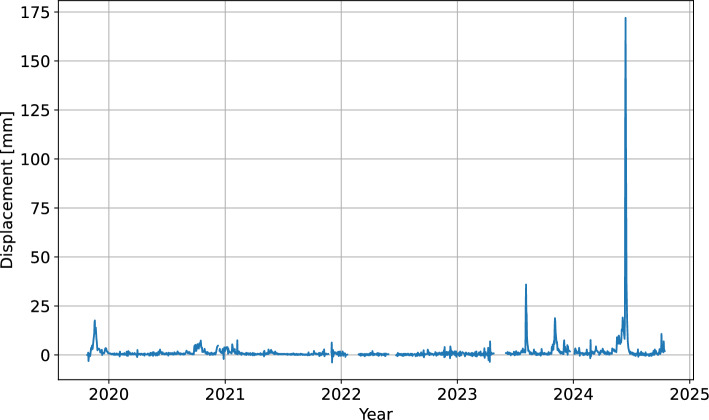
Time series of daily displacement for the bottom part of the Urbas landslide.

A time series of daily horizontal displacements is available for the period between October 2019 and October 2024 (Fig. 3). The raw time series contains 1562 data points representing the cumulative displacement at station GNSS4. There are in total 63 gaps in the data. The majority of them represent a day or two of missing data, which were mostly caused by the lack of sufficient sunlight to power the station. There are also seven gaps in the extent of a week or more, which were caused by instrument failure. Based on the analysis of displacement gradients on both sides of the gaps, up to six consecutive missing days were imputed using piecewise cubic Hermite interpolation, while the rest was not used for modeling. Finally, the cumulative displacement time series was differenced to obtain the daily prediction targets for model training and validation (Fig. 4). Due to the limited precision of GNSS measurements, small negative displacements were also present in the data. They occured during the less active periods when the actual landslide movement was close to the detection threshold.

#### Rainfall

Precipitation is a potentially very localized meteorological event, especially in mountainous areas where ridges act as natural barriers for low-altitude currents. To get reliable precipitation data for the Urbas microlocation, a stationary rain gauge was installed on the main body of the landslide (Fig. 1). An additional portable rain gauge was used for measuring precipitation in the landslide hinterland, but it was removed after a major malfunction at the end of 2023. Both devices recorded rainfall in hourly intervals. The stationary gauge worked quite reliably, and only had a single 10-day failure in December 2019. The gap was filled with the data from the portable gauge, which functioned properly at that time. The linear correlation of the two gauge time series for the whole observation period was $r=0.61$, indicating the local variability of precipitation even over short distances.

**Fig. 5:**
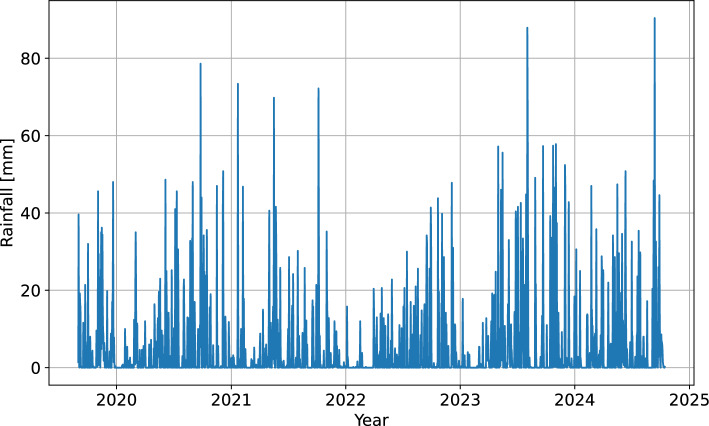
Time series of total daily rainfall for the Urbas landslide area.

Hourly precipitation data were summed to obtain the daily values (Fig. 5). However, there may be important differences between the effects of a highly concentrated downpour and a daylong drizzle that result in the same total daily rainfall. For this reason, the standard deviation of hourly values within the same day was included as additional feature for model training.

#### Groundwater

Groundwater levels are measured with hourly resolution at multiple locations on the landslide (Fig. 1). In a previous study by Peternel et al. ^44^, a correlation analysis was performed between the daily Urbas landslide displacements and the average GWL change over the past *N* days. The correlation $r=0.65$ was found for $N=5$ between the GNSS4 displacements and GWL data from the closest metering location P3. The correlation was slightly lower for uphill piezometer locations P1 and P2. In our study, the available time span of the GWL time series P1–P3 was more than twice compared to that in the previous study. This time, the highest direct correlation with GNSS4 displacements ($r=0.71$) was found for the daily GWL fluctuation at P1, which was therefore used in the prediction model.

**Fig. 6:**
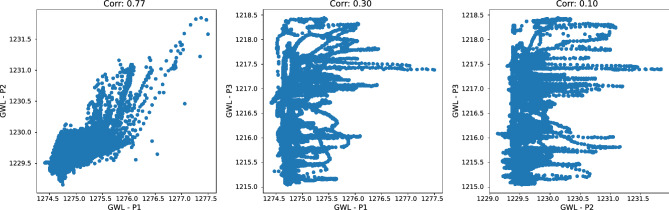
Correlation plots for GWL time series at piezometer locations P1 and P2 (left), P1 and P3 (middle), and P2 and P3 (right).

The GWL time series were not complete – P1 and P3 contained an identical 24-day gap between September and October 2021, while P2 contained several shorter and two longer gaps (47 and 74 days) for a total of 185 missing days. To impute the missing data, we have first calculated pairwise correlations of GWL time series for different lags *l* (Fig. 6).

**Fig. 7:**
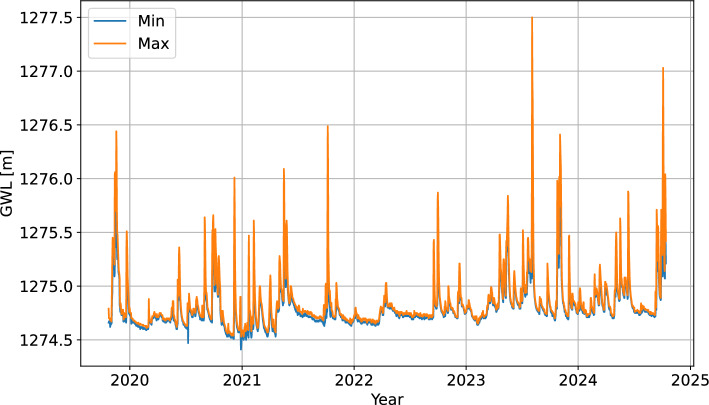
Time series of minimum and maximum daily GWL at the piezometer P1 location.

A relatively strong correlation $r=0.77$ was found between P1 and P2 at a 4-hour lag, while P3 was weakly correlated with the other two locations ($r=0.10$ and $r=0.30$). As there was no temporal overlap in the gaps within the time series P1 and P2, we have utilized the data from P2 for model-based imputation of P1, which initially had fewer missing data points. A linear regression model was constructed between P1 and P2, and used to impute the missing values in P1. The minimum and maximum GWL at P1 within a single day were used as aggregate daily predictors (Fig. 7).

#### Snow depth

**Fig. 8:**
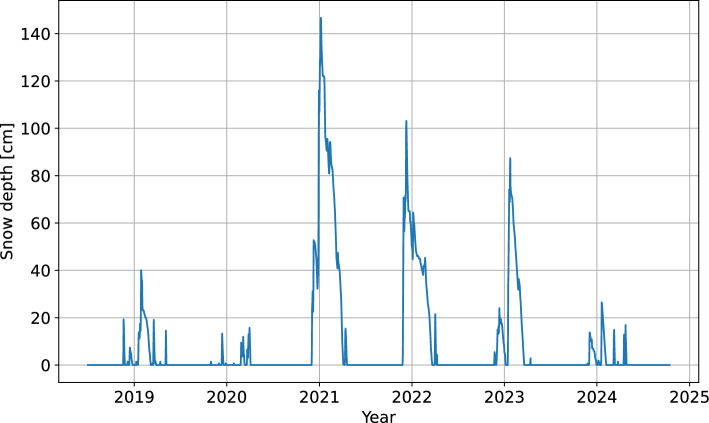
Snow depth time series at the weather station Javorniški Rovt (wider Urbas area).

The measurements of snow cover are not conducted on the Urbas landslide directly. Image-based estimation of snow depth was available from a nearby Čikla weather station, which is located at a similar altitude about 500 meters to the west of Urbas. However, the data collection only started in October 2022, so the time series was shorter than two years. There was also a gap of 73 missing days between the end of January and the start of April 2023, when the presence of snow is common in the area. For this reason, the complete and more accurate snow depth time series from the national precipitation station on Javorniški Rovt was used as proxy data for snow conditions in the wider Urbas area (Fig. 8). The station Javorniški Rovt resides at an altitude of 939 m, which is approximately 300 meters lower than the Urbas landslide. The distance from the landslide to the station is approximately 3.5 km in the north-west direction. The data from Javorniški Rovt can be obtained from the publicly available archive of the Slovenian Environment Agency.

#### Temperature

The Čikla weather station has been measuring the temperature in 30 minute intervals since August 2021. Occasional breakdowns of the sensor resulted in erroneous readings and gaps in the time series. The data were manually cleaned up and imputed using the temperature time series from two nearby national weather stations Planina pod Golico and Zelenica. The station Planina pod Golico lies at 957 m above sea level, which is approximately 300 m lower than the Urbas landslide, while Zelenica is located at an altitude of 1534 m, which is around 300 m higher than Urbas. The first station is 7 km to the northwest of Urbas, while the second station is 7.5 km to the east of it. Unlike precipitation, the temperature patterns are much more consistent regionally and change gradually with the elevation. The relative arrangement of weather stations and the landslide allowed reliable modeling of temperature conditions on the landslide.

**Fig. 9:**
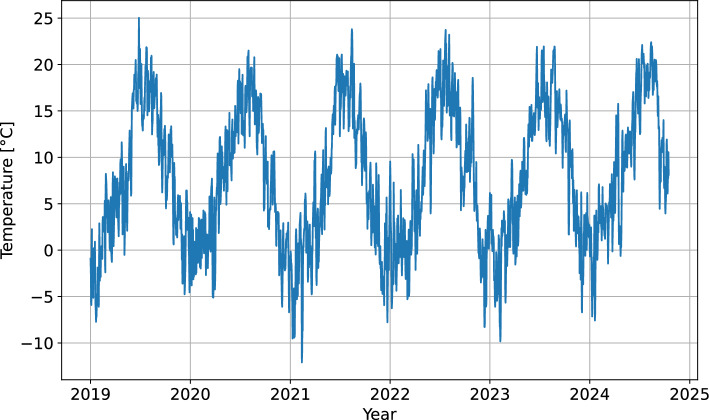
Time series of mean daily temperature for the Urbas landslide area.

In order to be compatible with the two auxiliary time series, the Čikla time series was first resampled to hourly resolution and aligned to whole-hour time points by linear interpolation. Two linear regression models were trained next, one for the typical meteorological conditions (i.e., lower temperatures at higher altitude of Zelenica) and another one for the cases of thermal inversion. The $R^2$ was 0.86 for the regular model and 0.94 for thermal inversion model, so both models were used to impute the missing hourly values in the Čikla time series. The choice of model for each data point depended on the relationship between the corresponding temperatures at the two weather stations. The reconstructed time series was finally aggregated into a daily one by computing the mean daily temperature (Fig. 9).

#### Earthquakes

Slovenia lies in a seismically active area, and currently has an established network of digital seismic observatories. In the period 2020-2022, the network registered around 2000 earthquakes yearly with the epicenter on Slovenian territory^50^. While truly devastating earthquakes are quite rare (only three cases with fatalities since 1895), there are between 30 and 40 earthquakes per year with a magnitude equal to or higher than 2 on the moment magnitude scale (Mw). They were included in this study to evaluate their potential impact on Urbas landslide dynamics. The effect of an earthquake on a landslide depends on the intensity of shakes reaching the observed area, which can, however, depend on a complex combination of geological conditions between the earthquake location and the landslide.

There is no dedicated seismological equipment installed in the landslide area, and the nearest seismic observatory Gorjuše is 16.5 km away. Unfortunately, the public archive of earthquake records^50^ does not provide the exact magnitudes registered at Gorjuše metering point for each earthquake event. For the purposes of this study, three earthquake attributes were therefore used as proxy indicators: the magnitude, the distance of epicenter from the landslide, and the estimated depth of the hypocenter. Because the magnitude is expressed on a logarithmic scale, its exponentiated value was used as input feature for the prediction model. The time of the earthquake was rounded down to the nearest whole hour. In case of overlapping narrowly separated aftershocks, the one with the highest magnitude was retained. The same rule was applied for the aggregation of hourly data into daily data.

### The proposed landslide model

In this study, a displacement forecasting model is proposed for the lower part of the Urbas landslide, its most active section and a potential source of debris flow. The model is based on a neural network that incorporates autoregressive and covariate structures as two separate branches. Both branches are implemented as convolutional neural networks. The convolutional architecture was selected for its ability to capture short-term correlations and causal relationships, which are characteristic for the observed toe of the Urbas landslide that responds rapidly to sustained rainfall and GWL fluctuations. Compared to recurrent neural networks, the training of CNNs is also considerably faster and more stable.

Two variants of the model are used – a single-task model, which predicts only daily displacements, and a multi-task model with two auxiliary tasks. Multi-task learning is a machine learning paradigm in which a model is trained to perform two or more related prediction tasks simultaneously using the same inputs. A multi-task model typically consists of a shared backbone that extracts general features and task-specific heads that map these features to individual predictions. It has been shown that multi-task learning can improve the performance on individual tasks by leveraging information across tasks^51,52^.

In the proposed multi-task displacement model, the first auxiliary task is to predict the maximum GWL change on the day of the forecast. GWL fluctuations are strong indicators of landslide velocity changes, but the actual GWL values are not available at prediction time and cannot be used directly as inputs. However, they can be estimated from recent GWL history and rainfall forecasts and used as auxiliary prediction targets. By learning to predict GWL change, the model is encouraged to extract features that are also informative for the landslide displacement prediction.

The second auxiliary target is a binary classification of whether the expected displacement will exceed a predefined warning threshold. The aim of this task is to focus on accurate detection of specific points in landslide activity, which is important for potential integration into a LEWS. It also acts as a form of regularization, reducing the risk that the training process is dominated by large residuals.

**Fig. 10:**
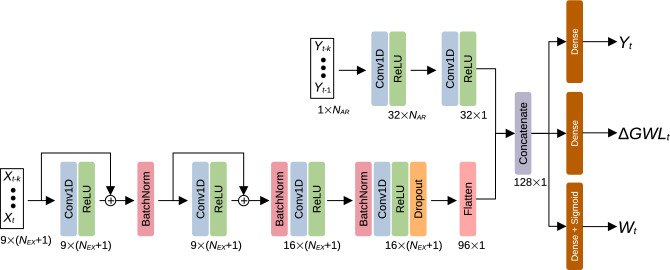
The proposed neural network model for landslide displacement prediction.

Figure 10 shows the architecture of the proposed multi-task model. The architecture of the single-task model is essentially the same, but uses only the output $Y_t$. The autoregressive component of landslide displacement is modelled by the upper branch in Fig. 10. It accepts a sequence of the last $N_{AR}$ daily displacements $Y_{t-k}~(k=1,\ldots ,N_{AR})$, and sequentially applies two 1D convolutions along the temporal dimension to extract a latent representation of recent landslide displacement trend. The first convolution uses kernel size 3 with zero padding, while the second convolution aggregates across the complete input sequence using kernel size $N_{AR}$ with no padding.

The bottom branch in Fig. 10 processes the recent $N_{EX}$ lagged values of *M* exogenous covariates $X^{(i)}~(i=1,\ldots ,M)$. It first extracts temporal covariate features using two residual convolutional blocks, where 1D convolutions with kernel size 3 are applied along the temporal dimension to each covariate channel separately. This is followed by a pair of cross-channel 1D convolutions (kernel sizes 1 and 3), which extract higher level features representing covariate interactions. Batch normalization is applied between every pair of convolutions to stabilize training, and a dropout layer with rate 0.5 is used as a regularizer to mitigate model overfitting. The extracted autoregressive and covariate features are finally concatenated and passed to three regression heads for displacement, GWL change and threshold warning prediction.

Landslide triggering factors that are recognized in the modern landslide studies mainly refer to precipitation^53^, and less to other factors as fluctuations in reservoir water level^54^, snowmelt^55^ or earthquakes^56^. In this study, we tested the potential impact on increased landslide activity considering all available datasets, namely the amount of precipitation (RainTotal), rainfall intensity (RainStd), snowmelt (combining SnowDepth and Temperature), groundwater level fluctuation (combining GWLMin and GWLMax), and the impact of earthquake on hillslope stability (combining EarthquakeDistance, EarthquakeMagnitude and EarthquakeDepth). The standard deviation of hourly rainfall was included as a proxy to account for potentially different hydrological effects of concentrated rain showers and daylong precipitation. The presence of snow on the landslide, indicated by the snow depth variable, can influence the slope dynamics by increasing the load and changing the timing and efficiency of groundwater infiltration. The temperature was included as a potential cofactor in snowmelt and a seasonal indicator. The daily maximum and minimum GWL were included as separate features to capture within-day and day-to-day fluctuations that are associated with accelerated landslide movement. The earthquake magnitude, epicentral distance and hypocentral depth were selected as proxy features that are commonly linked to perceived macroseismic intensity^57^. Table 2 provides the properties of all used daily covariates.

**Table 2:** Aggregated and derived features used as exogenous covariates in prediction models for landslide displacement.

Feature	Unit	Range of values	Description
*RainTotal*	mm/m$^2$	[0.0, 90.4]	Total amount of daily rainfall
*RainStd*	/	[0.0, 6.98]	Std. dev. of hourly rainfall
*SnowDepth*	cm	[0.0, 146.7]	Depth of snow
*Temperature*	$^\circ$C	$[-12.1,23.8]$	Mean daily temperature
*GWLMin*	m.a.s.l.	[1274.41, 1276.75]	Minimal daily groundwater level
*GWLMax*	m.a.s.l.	[1274.53, 1277.50]	Maximal daily groundwater level
*EarthquakeDistance*	km	[0.0, 195.3]	Distance of earthquake epicenter
*EarthquakeMagnitude*	/	$[0.0,10^4]$	Exponent. earthquake magnitude
*EarthquakeDepth*	km	[0.0, 21.0]	Depth of earthquake hypocenter

**Fig. 11:**
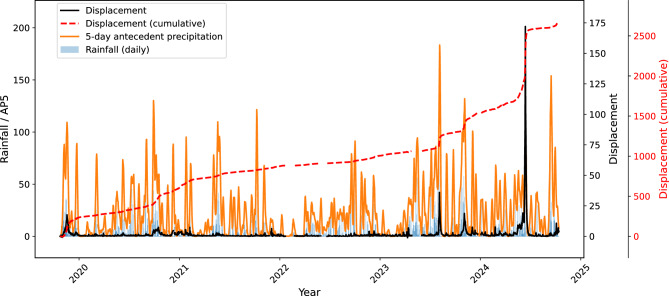
The overlay of daily rainfall, 5-day antecedent precipitation and landslide displacements, indicating step-like responses to intense and extended rainfall events. The curves have been smoothed for clearer view.

**Fig. 12:**
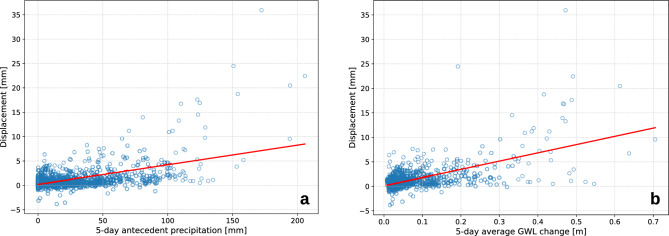
Correlations between landslide displacements and **a** 5-day antecedent precipitation and **b** Average 5-day antecedent daily GWL change in the training set.

Precipitation is assumed to be the main driver of Urbas acceleration, which can be confirmed by visual inspection of Fig. 11. The correlation between the displacements and 5-day antecedent precipitation in the training data was $r=0.55$ (Fig. 12a). Adding the rainfall variability (RainStd) to the model accounted for additional $6 \%$ of displacement variance, where more temporally concentrated rainfall was associated with reduced displacements. The 5-day average daily antecedent fluctuation of GWL was also moderately correlated ($r=0.62$) with the displacements (Fig. 12b).

**Fig. 13:**
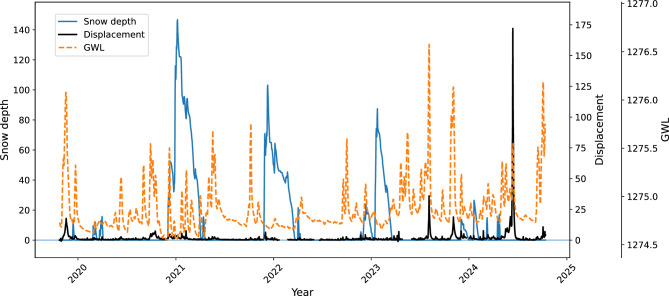
The overlay of snow depth, maximum GWL and landslide displacements. The curves have been smoothed for clearer view.

Figure 13 shows the correspondence between the estimated depth of snow cover, maximum GWL, and daily displacements in the toe of Urbas. The statistical analysis revealed that there was a weak ($r=0.18$) but reliable ($n=448$) positive correlation between snow depth and daily displacements on snow days. A weak positive correlation ($r=0.19$) between melt rate and displacements on melt days ($n=344$) was also detected.

After accounting for precipitation effects, there was no significant partial correlation between the displacements and the earthquake intensity, which was estimated as the exponentiated magnitude divided by squared epicentral distance and normalized hypocentral depth. A very weak positive correlation ($r=0.13$) was found between the displacement and earthquake intensity ranks during dry periods.

While the determined correlations are informative regarding the potential importance of features, they may overlook non-linear and lagged interactions, which can be captured by the model. We have therefore included all features in the base model and performed the ablation study to assess their importance for model performance.

When used for the training of neural network models, the covariate time series were normalized. To make predictions for some time point *t* during model training, the lagged values $X^{(i)}_{t-k}$ for $k=1,\ldots ,N_{EX}$ were used for all covariates. Additionally, the contemporaneous values $X_t$ of rainfall, snow depth, and temperature were used as inputs, since their predicted values (e.g., a weather forecast) can be used in the inference phase. For other covariates, the values from the previous time step were repeated as inputs at time *t* since their future values are not available in the inference phase. While hypothetical values of these variables could be used to perform scenario-based forecasting, we do not consider such model application in this study.

### Training and evaluation

The time series were first split into training and test sets. The data up to the end of March 2024 were used for fitting the models, while the rest was used for evaluation. A six-month period between October 2023 and March 2024 was used separately as a validation set for optimizing the architectures and training configurations of the neural network models. This division of data corresponded roughly to the 80/10/10 ratio commonly used in machine learning applications, and also ensured that every subset included periods of increased landslide activity. Once the models were selected, the validation set was repurposed for the final model training in order to maximize the amount of available training data.

The training of a neural network is an iterative process in which a batch of training examples with known target values is presented at the network input. This initiates the forward computation that generates the predictions at the network output. The loss function that reflects the closeness of the predictions to the actual values is evaluated next. This is followed by a network update phase in which the network parameters are adjusted in order to improve the predictions in the next cycle. The parameter updates are computed by an optimizer – a procedure based on gradient descent for finding the minima of the loss function. The two most common optimizer choices in modern machine learning are Adam and RMSProp, which dynamically adapt the stepsize of parameter updates. The training typically terminates after a certain number of epochs (i.e., presentations of the whole training set to the network) or when the loss stops to improve. For efficient learning, a proper configuration of training hyperparameters must be chosen.

The architecture (number and size of layers) and hyperparameters (learning rate, batch size, and number of epochs) were tuned on a validation set using grid search. The final configuration for the proposed model was as follows: the Adam optimizer was used with the default parameter values, the learning rate was $5\times 10^{-5}$, the batch size was 1024, and the maximum number of training epochs was 5000. The mean squared error (MSE) was used as a loss function for the landslide daily displacement prediction and, in the multi-task model, the prediction of *GWLMax* change.

In the multi-task model, the weighted binary cross-entropy (BCE) loss was additionally used for the auxiliary task of predicting the above-threshold landslide activity. Here, the output target was set to 1 only if the previous displacement was below a given threshold *T*, and the next displacement was above *T*. In all other cases, including repeated displacements above the threshold, the target for the warning output was set to 0. This design encouraged the model to predict accurately the first occurrences of increased landslide movement following the periods of below-threshold activity. The weight of positive samples was set to $w=30$, as this was approximately the ratio of negative vs. positive samples in the training dataset. The combined loss for multi-task training was then defined as:

1 $$\begin{aligned} L=(1-u_1)\cdot (L_{Y} + u_2\cdot L_{GWL}) + u_1\cdot L_{W}, \end{aligned}$$

where $u_1=0.9$ and $u_2=500$ were used to balance the contribution of losses. The displacement threshold was empirically set to $T=2$ mm as an early indicator of potential landslide acceleration, intended to support timely situation assessment by a human supervisor. The chosen value is above the estimated measurement error, and was verified to ensure enough positive cases in the training (45), validation (13) and test sets (11) for effective learning in an otherwise highly imbalanced prediction task.

The performance of the proposed model was compared with the following reference models:

- a naive model,
- a linear regression model,
- an ARIMAX model, and
- a gated recurrent unit (GRU) neural network model.

All of the models used the same history window widths $N_{AR}=5$ days for the displacements and $N_{EX}=5$ days for the covariates. The appropriateness of using $N_{AR}=5$ was selected based on the results of the partial autocorrelation function (PACF), which showed significant ($\alpha =0.05$) PACF values of displacements up to lag 2 with borderline significant remaining autocorrelation at lag 6. The suitability of window width $N_{EX}=5$ for past covariate values was confirmed based on the Pearson correlation between the displacements and total rainfall within the window, which peaked at 5 days.

The naive model always used the last known displacement value as prediction, which in many cases presents a solid reference, especially during the periods of linear landslide trend. Its predictive value for an early warning system is zero, however, since it will only predict the increased landslide activity after it was already observed.

The linear regression model used the lagged displacements and covariates as independent variables in a linear equation for displacement. With the selected window widths, the total number of estimated coefficients, including the intercept, was 54.

ARIMA (AutoRegressive Integrated Moving Average) is a statistical model used for time-series forecasting based on its own past values, past errors, and the use of differencing to make the series stationary. The ARIMAX model extends ARIMA by including covariate time-series as exogenous explanatory variables. We employed a non-seasonal ARIMAX model with an autoregressive order of $p=5$ and a corresponding number of lags for the exogenous predictors. The Dickey-Fuller test rejected the null hypothesis of non-stationarity for the daily displacement time series, suggesting the use of differencing order $d=0$. The moving average order was similarly set to $q=0$, which was determined by using the Ljung-Box test to check for significant autocorrelation of residuals on the training set. The final model was fitted using the Broyden-Fletcher-Goldfarb-Shanno (BFGS) optimization method.

GRU is a type of recurrent neural network for processesing sequential data^58^. At every time-step it accepts the current value of the target and a vector of contemporary covariate values, and outputs the prediction of the next target value. The architecture of the GRU is specifically designed for efficiency by addressing the vanishing gradient problem that impedes the flow of information during training. The basic building block of a GRU is a cell, which extracts feature vectors of given size from the input and uses them to update the hidden context for future inputs. Cells can be stacked into multiple layers to extract hierarchical features. In our experiments, a three-layer GRU was used with the hidden representation size of 128. The training sequences for GRU were generated similarly to those for the proposed model, except that the lagged covariate and autoregressive values were combined into a single vector at each time step. A training sequence for predicting the displacement at time step *t* therefore contained $N=5$ vectors of preceding covariate and autoregressive values at time steps $t-N,\ldots ,t-1$, as well as an additional time step containing the current values for rainfall, snow depth, and temperature at *t*. The input values for other covariates and displacement at time step *t* were repeated from those at time step $t-1$. The RMSProp optimizer was used with learning rate $5\times 10^{-6}$, batch size 1024, and 5000 training epochs, as it demonstrated more stable convergence than Adam.

The performance of models on the test set was evaluated according to two criteria – the mean absolute error (MAE) for displacement predictions, and the $F_1$ score for detecting all upward crossings of the displacement threshold. In the latter case, we observed the timepoints where the actual displacements or predictions went from below threshold to above threshold. The true positive case was registered if both the actual landslide movement and the prediction crossed the threshold, otherwise the case was marked as a false negative or a false positive.

## Results and discussion

In this section, we report and discuss the results of experiments with the employed models for Urbas landslide displacement prediction. We also describe the findings of the ablation study, which was realized by removing predictors from the model to determine their contribution to landslide displacement.

The experiments were conducted on a personal computer with AMD Ryzen 9 7950X3D CPU, 64 GB of system memory, and an Nvidia GeForce RTX 4090 GPU with 24 GB of VRAM. Python programming language was used for implementation, where scikit-learn library was used for linear regression, statsmodels library for the ARIMAX model, and Pytorch for the neural network models.

### Model performances

**Fig. 14:**
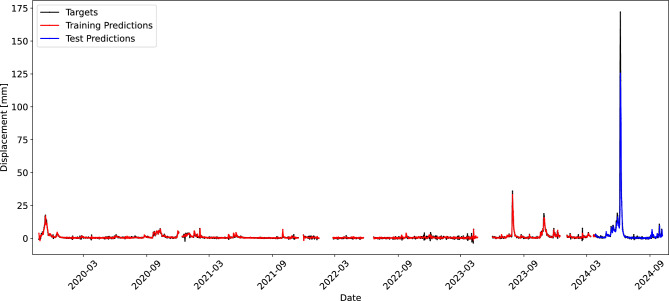
Target values (black) and predictions made by the proposed CNN model on the training set (red) and the test set (blue).

**Fig. 15:**
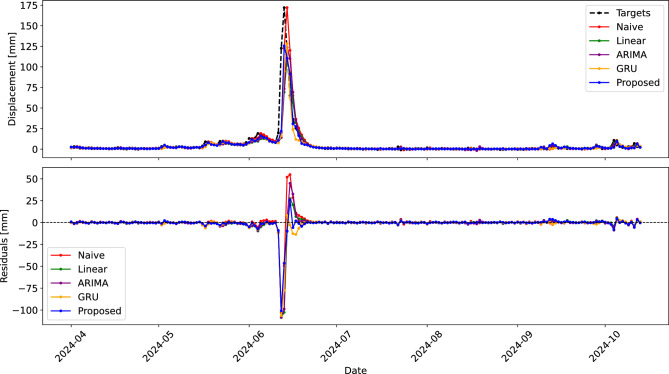
Predictions and residuals of different models on the test set.

Table 3 shows the performance results for the tested models in terms of the achieved MAE and $F_1$ score. For neural network models, the median and the best MAE and $F_1$ are reported out of 15 training repetitions. Figure 14 shows predictions of the proposed model on both the training and the test dataset, while Fig. 15 visualizes the predictions made by different models on the test set.

**Table 3:** Test set performance of models for landslide displacement prediction.

Model	MAE	$F_1~(P/R)$
Naive	2.59	0.000 (0.000/0.000)
Linear regression	2.41	0.571 (0.600/0.545)
ARIMAX	2.46	0.632 (0.750/0.545)
GRU median	2.03	0.250 (0.400/0.182)
GRU best	2.00	0.267 (0.500/0.182)
Proposed single-task median	1.88	0.316 (0.375/0.273)
Proposed single-task best	1.83	0.500 (0.556/0.455)
Proposed multi-task median	1.85	0.526 (0.625/0.455)
Proposed multi-task best	1.79	0.636 (0.636/0.636)

The results show that the lowest displacement prediction errors were achieved by the proposed single-task and multi-task neural networks. They were followed in forecasting accuracy by the GRU model, which was very sensitive to initial conditions and hyperparameter settings. In our experiments, GRU failed to converge in approximately one out of four training attempts even after careful tuning of the architecture and learning rate. Despite its simplicity, the linear regression model performed better than ARIMAX in terms of displacement prediction accuracy. As expected, the naive prediction performed the worst, though it still served as a strong baseline.

Not surprisingly, the naive model proved to be useless as a base for an early warning system. The overall best $F_1$ for warning prediction was achieved by the proposed multi-task model, which was not only substantially better than the single-task model in detecting the threshold crossings, for which it was trained through the auxiliary target, but had a lower MAE at the same time. The ARIMAX and linear regression models provided competitive $F_1$ scores, while the GRU model struggled to predict when the displacements would cross the warning threshold. Common to all models was that the precision was typically higher than the recall, meaning that the number of false negatives (i.e., instances where no warning was issued when it should have been) was higher than the number of false positives (i.e., sounding false alarms).

### Ablation study

The goal of the ablation study was to analyze the effects of removing predictors from the proposed multi-task model. The predictors were divided into four groups based on covariate time series they originated from in the following way:

1. covariates related to earthquakes, i.e., the magnitude, distance and depth of the earthquake;
2. covariates related to snowmelt, i.e., the depth of snow and the temperature;
3. covariates related to groundwater level, i.e., the minimal and maximal daily GWL;
4. covariates related to rainfall, i.e., the daily total and standard deviation of rain.

Table 4 shows which groups of covariates were used by reduced model versions M1–M5 of the complete base model M0, where model M5 is essentially a pure non-linear autoregressive model. Table 5 reports the achieved median MAE and $F_1$ out for 15 training invocations.

**Table 4:** Covariate groups used by models in the ablation study.

Model	Rainfall	GWL	Snowmelt	Earthquakes
M0	$\checkmark$	$\checkmark$	$\checkmark$	$\checkmark$
M1	$\checkmark$	$\checkmark$	$\checkmark$	✗
M2	$\checkmark$	$\checkmark$	✗	✗
M3	$\checkmark$	✗	✗	✗
M4	✗	$\checkmark$	✗	✗
M5	✗	✗	✗	✗

**Table 5:** Test set performance of progressively reduced multi-task models for landslide displacement prediction.

Model	MAE	$F_1~(P/R)$
M0	1.85	0.526 (0.625/0.455)
M1	1.88	0.476 (0.500/0.455)
M2	1.88	0.421 (0.500/0.364)
M3	1.85	0.421 (0.500/0.364)
M4	1.84	0.375 (0.600/0.273)
M5	1.82	0.000 (0.000/0.000)

**Fig. 16:**
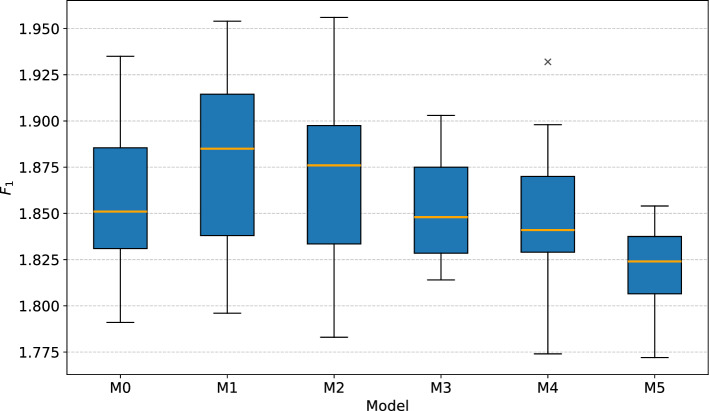
Box plots of displacement MAE for models used in the ablation study (lower is better).

Figure 16 shows the MAE box plots of the models. The normality of sample distributions and equality of their variances were checked with the Shapiro-Wilk and Levene’s test, respectively. The ANOVA test rejected the null hypothesis of no differences between the models’ performances ($p=0.007$). The Tukey’s HSD post-hoc test revealed statistically significant difference only between pairs of models M1-M5 and M2-M5, though they are of little practical importance.

An interesting result of the experiment is that the purely autoregressive model M5 achieved, on average, the lowest MAE. The analysis revealed that M5 accomplished this by modeling more accurately the non-linear receding phases of landslide movement, which behave quite predictably in the absence of additional precipitation. Since returning to normal movement typically takes more time than the acceleration, accurate predictions of landslide deceleration resulted in low overall error.

**Fig. 17:**
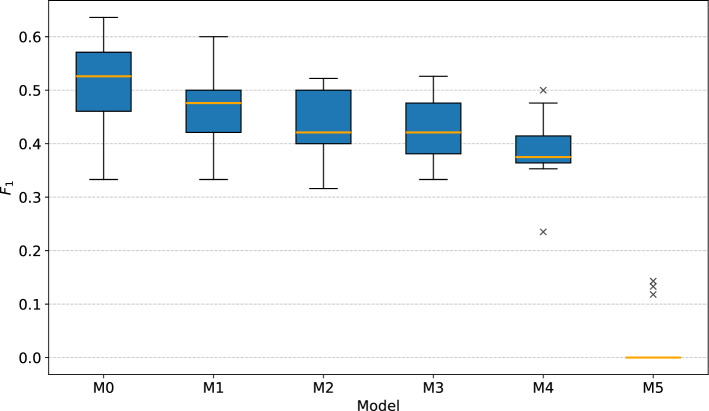
Box plots of threshold displacement warning $F_1$ for models used in the ablation study (higher is better).

On the other hand, the M5 model completely failed to capture any factors leading to landslide acceleration. This is evidenced by the plot of $F_1$ scores in Fig. 17, which renders the M5 model useless for LEWS. Because not all models’ results were normally distributed, the Kruskal-Wallis test was used to check for significant performance difference, and rejected the null hypothesis of no difference ($p=0.000$). The Dunn’s post-hoc test indicated significant difference between model M5 and all other models, as well as between the models M0 and M4. This suggests that precipitation, which is included in models M0-M3, is the primary initiator and the most relevant indicator of landslide acceleration. The inclusion of features related to snowmelt and earthquakes slightly improved the median case of threshold exceedance detection, but the differences in overall performance were not statistically significant.

#### Sensitivity analysis

**Fig. 18:**
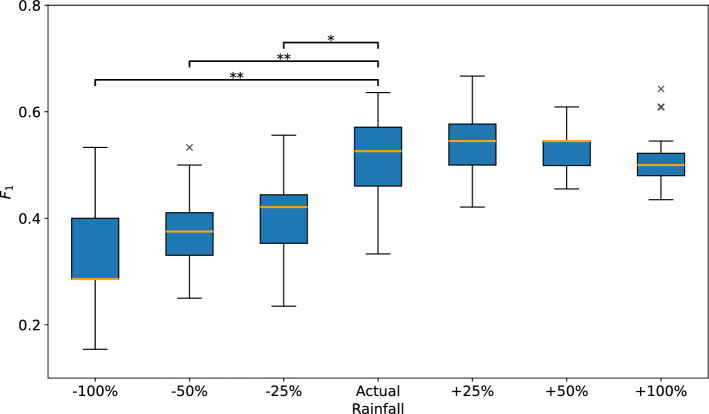
$F_1$ for threshold crossing detection with model M0 when using consistently biased estimates of rainfall amount on the day of prediction, simulating imperfect weather forecasts. Statistically significant differences between performances obtained with varying levels of bias are marked with horizontal bars above the box plots at significance levels $\alpha =0.05$ (*), and $\alpha =0.01$ (**).

Once put into operation, the model would have to rely on imperfect weather forecasts rather than actual, contemporaneous measurements of covariates. The purpose of the final experiment was to assess how sensitive the model is to inaccuracies in such covariate estimates. We have used model M0 for this experiment, and assumed an extreme scenario in which the rainfall forecasts were consistently biased by $25 \%$, $50 \%$, and $100 \%$ in both positive and negative directions. The results are presented in Fig. 18, which shows the test performance of 15 trained models when different levels of error are added to the actual amount of rainfall on the day of prediction. Friedman non-parametric test rejected the null hypothesis of no difference between the performances with different levels of error. Wilcoxon Signed-Rank test with Holm correction was used for pairwise comparisons.

No significant differences were found in the ability of the models to predict the crossing of displacement threshold when the actual rainfall value was increased by up to $50 \%$. On the other hand, underestimating the amount of next day’s rainfall led to significant reduction of threshold crossing detection. A possible explanation is that there is a threshold amount of precipitation required to trigger the initial landslide acceleration. By reducing the expected rainfall volume, the remaining effect may be too weak to push the predicted displacement above the threshold. On the other hand, the effects of increased rainfall values are not symmetric, since during rain showers much of the water runs off on surface without infiltrating the groundwater table. Another potential reason for the observed model behavior is that the on-site measurement equipment under-measures during heavy precipitation (e.g., due to saturation).

### Discussion

The study presented in this paper is the first attempt to build a data-driven ML-based model of lower part dynamics of Urbas, whose failure could result in a debris flow with catastrophic consequences for the endangered settlements. The obtained results support the assumption that the acceleration of Urbas is induced mainly by rainfall and related changes in groundwater level, since the removal of other covariates did not significantly reduce the model’s predictive ability. These findings agree with the site-specific engineering geological conditions and reasonably complement the existing knowledge on the dynamics of the Urbas landslide. The upper part of the landslide is covered by up to 20 m thick carbonate scree deposits^46^ and precipitations are entirely infiltrated through this permeable layer down to the contact with the Paleozoic clastic rocks. Both scree deposits that are covering the upper part of the landslide and the wider hinterland built of carbonate rock are the main landslide recharge areas^59^. In the upper part of the landslide, groundwater is deep, while at the lower edge of the scree deposits at the contact with weakly permeable clastic rocks, groundwater occurs close to and on the surface in the form of several springs and wetlands. At the eroded toe of the landslide the groundwater is shallow and consists of weathered clastic rocks mixed with scree. In this setting, rainfall events rapidly translate into groundwater level fluctuations, which reduce shear resistance and trigger accelerated movement. In addition, extended rainfall increases the discharge of the Bela stream and enhances fluvial erosion at the toe, further contributing to its acceleration.

Most of the existing literature on landslide displacement prediction relies on displacement forecast error as the main performance metric for comparing different forecasting models. The results of Urbas study demonstrate that models with comparative performance in terms of MAE can differ substantially regarding their ability to detect critical points in landslide evolution. The inverse also holds – a model with worse MAE can be more successful at discovering the threshold-crossing points. We plan to further investigate this property and devise additional performance measures relevant to practical aspects of models’ use in LEWS. While the current accuracy of predictions does not yet meet the operational requirements for LEWS, the model could be used to issue early warnings for a timely situation assessment by a human operator. The multi-task training paradigm showed promising results, and is also worthy of further investigation. While each landslide’s dynamics depends on site-specific geological and hydrometeorological settings, the proposed methodology is general and provides a basis for modeling other similar landslides.

Over an extended monitoring period, the landslide dynamics can change in locally constrained ways that can affect the accuracy of any model based on few measurement locations. Expanding the sensor network is important not only for providing more data, but also for introducing redundancy in cases of fallout, which are more frequent in remote and exposed mountainous regions. Additional monitoring equipment, consisting of a flow meter for measuring surface stream discharge and sensors for monitoring soil moisture, which are planned to supplement the existing set of monitored parameters on Urbas, will potentially contribute to improved model performance. As the volume of collected data continues to grow, more sophisticated machine learning models can be trained without overfitting. However, this study shows that the triggering mechanisms for the lower part of Urbas landslide have relatively short latency. Consequently, complex and hard-to-train recurrent neural network architectures become less necessary, favoring convolutional models capable of detecting short-term sequential patterns. We have demonstrated that such non-linear models outperform traditional and linear models.

There are several directions for further improvements. The present report addressed one-step-ahead forecasting of landslide displacements. Predicting the landslide evolution several days into the future would provide more time for preparation and allow the decision-makers to decide when to suspend the existing safety measures. However, multi-step displacement predictions typically have larger margins of error because the long-term weather forecasts used during inference are, by nature, also less reliable. The sensitivity analysis showed that misprediction of rainfall does not have a symmetric effect on the proposed model, which is more sensitive to underestimation than to overestimation. The analysis of rainfall forecast and its agreement with on-site measured precipitations could help tune the LEWS to its best performance.

The present approach used data imputation and aggregation of hourly sensor data into daily totals, which allowed us to directly compare the employed prediction models. However, data aggregation can obscure subtle patterns in covariate time series that could be useful for prediction. The advantage of the proposed deep neural network architecture lies in its ability to seamlessly accommodate multi-resolution inputs, enabling the processing of different time series in their native temporal resolutions.

## Conclusion

In the paper, a multi-task learning approach was proposed for a model that predicts daily displacements of Urbas landslide in north-western Slovenia. The proposed model is based on a convolutional neural network that uses as inputs the recent history of landslide movement and multiple time series of exogenous covariates corresponding to hydrometeorological and seismological conditions in the area. The network architecture features separate branches for processing the autoregressive and covariate components.

The comparison with naive, linear, ARIMA and GRU models showed that the proposed model outperformed them in terms of MAE. However, we also demonstrated that MAE is not a representative metric for potential integration of the prediction model in LEWS, where the focus is on detecting threshold-crossing events in landslide activity. The experiments revealed that achieving this goal does not necessarily align with the model’s raw displacement prediction accuracy. To address this, we employed a multi-task approach to model training, where the auxiliary tasks were to predict the GWL change and whether the displacement will exceed a warning threshold. This significantly improved the accuracy of threshold-crossing prediction compared to the single-task model, although further improvements are needed to achieve the low tolerance for errors in LEWS applications.

The ablation study confirmed the findings of previous geological surveys that extended rainfall is the main trigger of increased Urbas activity. The inclusion of indicators related to snowmelt and seismic activity provided small median improvements in threshold exceedance prediction, but the overall performance differences were not statistically significant, indicating lack of explanatory power for these predictors. The analysis of sensitivity to inaccurate precipitation forecasts resulted in asymmetric conclusions – underestimation of precipitation had an observable negative impact on model performance, while overestimation did not.

## Data Availability

The datasets generated during and/or analysed during the current study are available from the corresponding author on reasonable request.
